# Islets-on-Chip: A Tool for Real-Time Assessment of Islet Function Prior to Transplantation

**DOI:** 10.3389/ti.2023.11512

**Published:** 2023-10-11

**Authors:** Matthieu Raoux, Sandrine Lablanche, Manon Jaffredo, Antoine Pirog, Pierre-Yves Benhamou, Fanny Lebreton, Anne Wojtusciszyn, Domenico Bosco, Thierry Berney, Sylvie Renaud, Jochen Lang, Bogdan Catargi

**Affiliations:** ^1^ University of Bordeaux, CNRS, Institute of Chemistry and Biology of Membranes and Nano-Objects, UMR 5248, Pessac, France; ^2^ University of Grenoble Alpes, Clinique d’Endocrinologie, Diabétologie, Maladies Métaboliques, CHU Grenoble Alpes, U1055 INSERM, Grenoble, France; ^3^ University of Bordeaux, CNRS, Bordeaux INP, Laboratoire de l’Intégration du Matériau au Système, IMS UMR 5218, Talence, France; ^4^ Cell Isolation and Transplantation Center, Department of Surgery, Geneva University Hospitals, University of Geneva, Geneva, Switzerland; ^5^ Centre Hospitalier de Montpellier, Service d’Endocrinologie, Université de Montpellier, Montpellier, France; ^6^ Service d’Endocrinologie-Diabétologie, Hôpital St André, CHU de Bordeaux, Bordeaux, France

**Keywords:** islet transplantation, transplant assessment, electrophysiology, diabetes mellitus, islet, multielectrode array

## Abstract

Islet transplantation improves metabolic control in patients with unstable type 1 diabetes. Clinical outcomes have been improving over the last decade, and the widely used beta-score allows the evaluation of transplantation results. However, predictive pre-transplantation criteria of islet quality for clinical outcomes are lacking. In this proof-of-concept study, we examined whether characterization of the electrical activity of donor islets could provide a criterion. Aliquots of 8 human donor islets from the STABILOT study, sampled from islet preparations before transplantation, were characterized for purity and split for glucose-induced insulin secretion and electrical activity using multi-electrode-arrays. The latter tests glucose concentration dependencies, biphasic activity, hormones, and drug effects (adrenalin, GLP-1, glibenclamide) and provides a ranking of CHIP-scores from 1 to 6 (best) based on electrical islet activity. The analysis was performed online in real time using a dedicated board or offline. Grouping of beta-scores and CHIP-scores with high, intermediate, and low values was observed. Further analysis indicated correlation between CHIP-score and beta-score, although significance was not attained (R = 0.51, *p* = 0.1). This novel approach is easily implantable in islet isolation units and might provide means for the prediction of clinical outcomes. We acknowledge the small cohort size as the limitation of this pilot study.

## Graphical Abstract

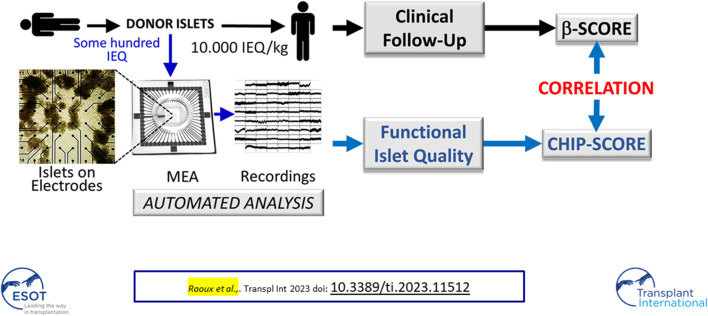

## Introduction

The incidence of type 1 diabetes is increasing worldwide and pancreatic islet replacement has emerged as a therapy, especially in the case of recurrent severe hypoglycemic events [1–4]. Transplantation of donor islets, obtained by mechanical and enzymatic dissociation of the pancreas, provides sustained improvement of glycemic control with an efficient prevention of severe hypoglycemia in the large majority of recipients, thus improving patients’ quality of life [5]. Moreover, islet transplantation has been demonstrated to prevent the progression of chronic diabetes complications [6–8].

Islet graft function can be assessed as clinical outcome by several methods, such as the β-score [9] or the Igls criteria [10]. The islet grafts’ potency can be assessed *in vivo* by transplanting a set fraction of the islet preparation into immunodeficient rodents, however, the read-out is retrospective [11, 12]. We are still lacking predictive criteria for evaluating islet quality immediately prior to transplantation [12], an issue that has been called for early on [13]. This issue will also be of considerable importance for potential future therapies using stem cell-derived surrogate islets [12].

Therefore, we investigated whether donor islet quality could be ranked according to their electrical activity. Considerable knowledge in the electrophysiology of human islets has been acquired during the last decade and allows to define meaningful electrophysiological parameters to evaluate their function and establish a ranking score [14]. Indeed, changes in ion fluxes are the first integrative signals of islet activity. Slow potentials (SPs), as recorded here by dynamic multi-electrode arrays (MEAs), reflect the physiological important coupling between islet β-cells, are tightly linked to insulin secretion and exhibit the same biphasic profile, a hallmark of islet activation [15, 16]. Moreover, these SPs are regulated by relevant hormones, such as adrenalin or incretins, and are deteriorated during aging and glucotoxic condition [15, 17]. Finally, these recorded electrical activities can regulate glucose homeostasis *in silico* in the FDA-approved simulator of glucose homeostasis in type 1 diabetes patients [18, 19]. In view of these characteristics of the recorded electrical islet signatures, we hypothesized that a detailed and dynamic electrophysiological analysis may reflect donor islet quality. Moreover, the use of extracellular electrophysiology applied here only requires routine expertise in cell culture, which is available in most clinical laboratories. The analysis of recorded data can either be automated and performed online or offline with commercial software or after electronic data exchange with expert groups.

## Materials and Methods

### Study Design and Scores

Islet CHIP (authorization number NCT03067324) was a pilot study derived from the clinical islet transplantation STABILOT randomized control trial (authorization number NCT02854696) [20]. Eight islet transplant recipients were investigated (see Table 1; Supplementary Table S1) from the study. Thirty patients were initially eligible for the study but 22 had to be excluded subsequently (donor research opposition, 11; receiver’s consent unknown, 5; COVID-related problems in patient follow up, 4; logistic problems, 2). For each recipient, an aliquot of islets (1,000 IEQ) was sampled from the islet preparation used for the first infusion and examined for glucose-stimulated insulin secretion (GSIS) and electrophysiology. Recipients were transplanted as described [20]. The immunosuppression protocol was as follows: thymoglobulin administration 2 days before islet transplantation; 1 hour before the first thymoglobulin infusion, 2 mg/kg methylprednisolone was administered intravenously and pentoxifillin (400 mg twice a day for 5 days) was started. A second thymoglobulin infusion (1 mg/kg body weight) was administered the day before the transplantation, a third thymoglobulin infusion (1.5 mg/kg body weight) was administered on the day of transplantation and again 2 days after transplantation. Etanercept (50 mg intravenously) was administered on the day of islet infusion, and subsequently administered subcutaneously (25 mg) on days 3, 7, and 10. Heparin (35 UI/kg) was injected into the portal vein just before islet infusion, followed by intravenous heparin infusion for 2 days, and finally subcutaneous application until day 8 after islet infusion. Tacrolimus (1 mg twice a day) was started and then adjusted according to residual tacrolimus blood concentrations with a target between 9 and 13 ng/mL for 3 months after transplantation, and subsequently decreased to a target between 6 and 10 ng/mL. Mycophenolic acid (1 g, twice daily) was administered the day before the first islet infusion. Detailed information is given in Supplementary Table S1.

**TABLE 1:** Characteristics of the study population (N = 8) and islet donors (N = 8).

A. Recipient population	
Baseline characteristics	Mean (SD)
Age (years)	48.3 (±4.8)
BMI (kg/m^2^)	22.8 (±2.5)
Daily Insulin Dose (UI/kg/day)	0.47 (±0.1)
Glycemia (mmol/L)	13.1 (±3.2)
HbA1c (%)	7.9 (±0.8)
(mmol/mol)	63.0 (±6.4)
Basal C-Peptide (ng/mL)	0.05 (±0.06)
**After the first islet infusion**	**Mean (SD)**
Daily Insulin Dose (UI/kg/day)	0.36 (±0.1)
Glycemia (mmol/L)	6.3 (±1.3)
HbA1c (%)	7.9 (±1.1)
(mmol/mol)	63.0 (±9.7)
Basal C-Peptide (ng/mL)	1.5 (±0.4)
**Before the 2nd islet infusion**	**Mean (SD)**
Daily Insulin Dose (UI/kg/day)	0.18 (±0.1)
Glycemia (mmol/L)	6.0 (±0.6)
HbA1C (%)	6.0 (±0.4)
(mmol/mol)	42.0 (±2.3)
Basal C-Peptide (ng/mL)	1.3 (±0.5)
**B. Donor population**	
**Baseline characteristics**	**Mean (SD)**
Age (years)	49.1 (±7.3)
BMI (kg/m^2^)	29.8 (±7.1)
GSIS Index	2.4 (±0.4)
GSIS with theophylline Index	6.0 (±1.7)
Purity (%)	73.0 (±21)

To rank patients’ clinical outcomes after islet transplantation, the β-score was used [9]. This score gives 2 points each for normal fasting glucose (≤5.5 mmol/L), HbA_1c_ (≤6.1% (43 mmol/mol)), stimulated and/or basal C-peptide (≥0.3 nM), and absence of insulin or oral hypoglycemic agent use. No points are awarded if the fasting glucose is in the diabetic range, HbA1c >6.9%, C-peptide secretion is absent on stimulation, or daily insulin use is >0.24 units/kg. One point is assigned for intermediate values. The graft function is considered optimal for a β score of 7 or 8, suboptimal for values between 6 and 4, and poor if 3 and lower. Clinical metabolic data were collected and β-scores were determined at inclusion in the study, 1 month after the first infusion, and before the second infusion (between 1 and 3 months after the first infusion). CHIP-scores from 1 (lowest) to 6 (highest) were attributed to donor islet preparations after exposing them to various physiological conditions (for details see Results).

### Human Islet Preparation

Human islets were isolated at the Geneva Cell Isolation and Transplantation Center from pancreata obtained from braindead multiorgan donors through the Swiss Transplant Agency and the French Biomedicine Agency (Agence de la Biomédecine). Islets were isolated using the automated method described by Ricordi et al. [6], with local modifications as previously reported [21] and glucose-induced insulin secretion (GSIS) measured as described [21]. GSIS is defined as the fold increase in static insulin secretion between 2.8 and 16.7 mmol/L of glucose (in the absence or presence of the cAMP raising agent theophylline). Detailed information is given in Supplementary Table S1.

### Electrophysiology

Aliquots sampled from islet preparations dedicated for transplantation were shipped to Bordeaux, seeded on multi-electrode arrays (MEAs, 60MEA200/30iR-Ti-gr, MCS, Reutlingen, Germany) coated with Matrigel (2% v/v; BD Biosciences, San Diego, CA, USA) by application in 10 µL and gentle concentric rotation in the middle of the MEA chip, and cultured at 37°C (5% CO_2_, >90% relative humidity) using CMRL-1066 medium (5.6 mmol/L glucose, 10% vol./vol. FBS, penicillin-streptomycin and L-glutamine) [15]. Solutions were replaced by pipetting during dynamic recordings. MEA recordings were performed at 37°C and pH 7.4 in solutions containing (in mmol/L) NaCl 135, KCl 4.8, MgCl_2_ 1.2, CaCl_2_ 1.8 (or zero, when indicated, to inhibit any electrical signals), HEPES 10 (pH 7.4 adjusted with NaOH) and glucose and drugs as indicated [22]. GLP-1 solutions (Bachem Bioscience, King of Prussia, PA, USA) were prepared *extempore*, adrenalin and glibenclamide were obtained from Sigma-Aldrich (St. Louis, MO, USA). Extracellular field potentials were acquired at 10 kHz, amplified and band-pass filtered at 0.1–3,000 Hz with a USB-MEA60-Inv-System-E amplifier (MCS; gain: 1200) controlled by MC_Rack software (v4.6.2, MCS) [15, 22, 23].

### Analysis

Dynamic electrophysiological recordings were either analyzed on-line in real time [23, 24] or off-line as described [15, 22, 25]. Correlation analyses were performed using SAS v9.4 (SAS Institute, Cary, NC, USA) and confirmed by SPSS® Statistics (IBM, New York, NY, USA). Plotting was performed using Prism 7.

## Results

**FIGURE 1:**
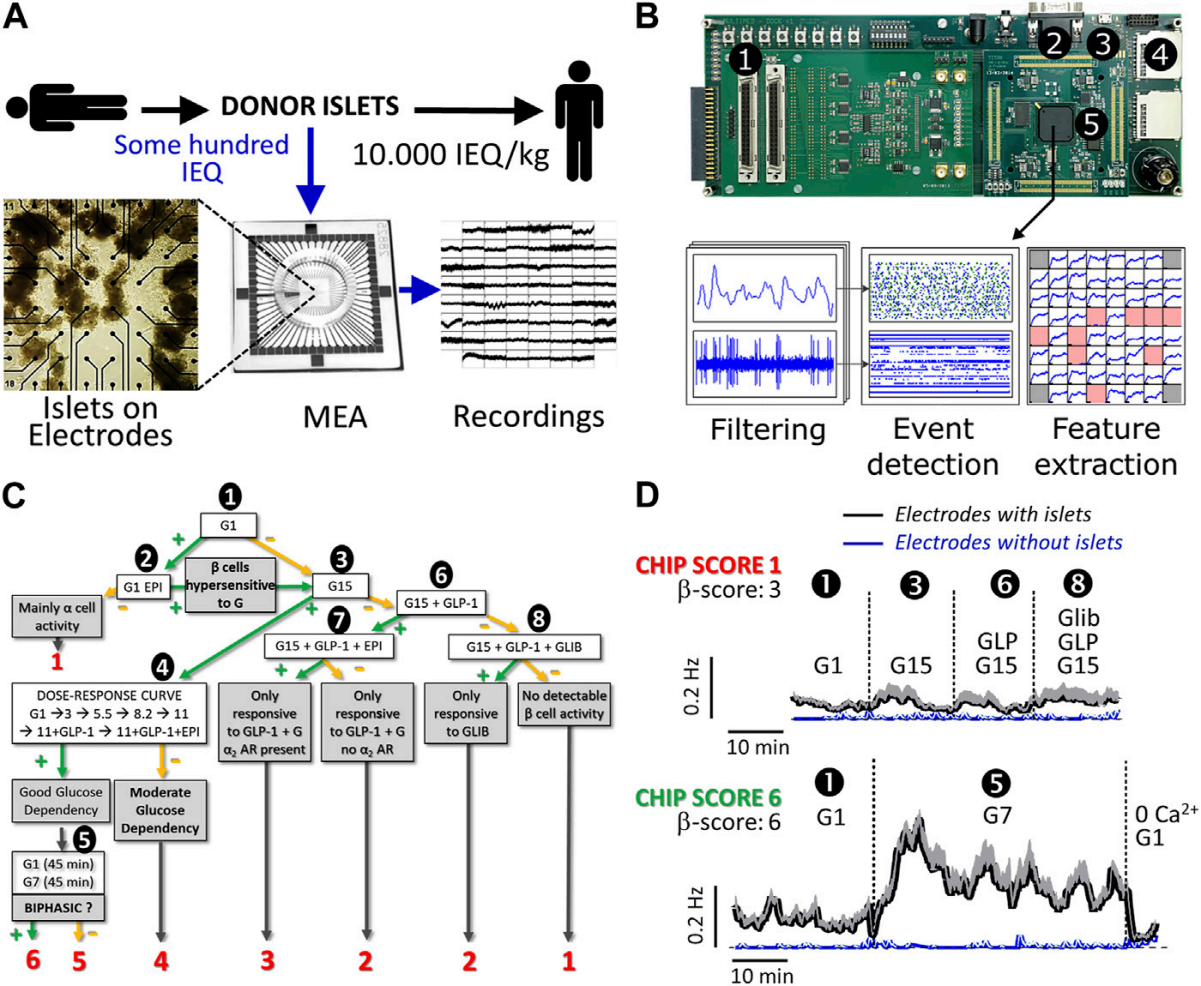
Flow process and examples of donor islet evaluation by micro-electrode arrays. **(A)** General work flow: a small number of donor islets were seeded on microelectrode arrays (MEAs) to record the electrical activity. **(B)** Custom electronics for real-time processing of islet signals. ① Signal inputs (x60); ② VGA monitor output for live display; ③ USB port for board configuration and recording control; ④ SD memory card slots for recording of islet cell signals and processed data; ⑤ Digital signal processing board for real-time filtering, event detection, and measurement of activity markers; **(C)** Algorithm for gradual islet ranking via CHIP-score; decision points are given in bold text and final ranking outcome from 1 to 6 in red. CHIP-score represents the highest rank attained by an islet preparation. Absence or presence of effect after test stimuli is indicated by—(green) or—(orange) symbols. First, the activity was recorded at 1 mmol/L glucose (G1, ①) when β-cells should be silent. If activity is present (>0.5 Hz), the use of epinephrine (EPI, 5 μmol/L, ②) permits the distinction between hyperactive islets (silencing) and islets containing mainly α-cells (enhanced activity). In that case, islets were not further analyzed. Subsequently, islets were exposed to 15 mmol/L glucose (G15, ③); if significant increases in slow potentials were observed, islets were submitted to a full physiological range of glucose concentrations (1, 3, 5.5, 8.2, 11 mmol/L) as well as GLP-1 (testing for incretins) and EPI (for silencing) ④. In the case of a dose-dependent glucose response, islets were tested for the presence of a biphasic response ⑤, a hallmark of islet activity. If islets did not respond to G15 (③), they are exposed to G15 in combination with an incretin (GLP-1, 50 pmol/L, ⑥). When the incretin had a stimulatory effect, a physiological stress hormone (epinephrine, EPI, 5 μmol/L, ⑦) was added to verify the inhibition, i.e., the presence of functional α_2_ adrenergic receptors (α_2_AR), as expected in β-cells. In case of absence of response to GLP-1, the sulfonyl urea glibenclamide was added (GLI, 100 nmol/L, ⑧) to test for the functional presence of K_ATP_ channels. Depending on the path in this decision tree and on the final point of arrival, the CHIP-scores indicated in red were attributed to each islet preparation. **(D)** Analysis of two donor islets (slow potential frequencies ±SEM) representative of the lowest and highest CHIP-scores (1 and 6, respectively). The test steps are given and numbers (①, ③, ⑥, ⑧ and ⑤) correspond to steps indicated in **(C)**. Means of recorded slow potential frequencies are given in black (SEM in grey) and noise (electrodes without islets) are given in blue (SEM in grey).

Islet signals were recorded using electrodes that measure extracellular islet field potentials due to ion channel activities. In our approach approximately 100 IEQ donor islets suffice to analyze their quality (Figure 1A) after seeding on a commercial microelectrode array (MEA); thus, only a minute aliquot of islets was necessary from a single donor as compared to the 10,000 IEQ per kilogram body weight required for transplantation. The recorded signals were amplified, digitized, and processed in real-time using dedicated hardware that applies a series of filters and detection algorithms to extract SP frequencies, which are representative of islet activity (Figure 1B) [15, 22]. The analysis can be performed directly online in real-time using custom electronics as given in Figure 1B, which provides automated filtering of recordings, detection of electrical signals (events) and feature extractions [23, 24].

As extracellular electrophysiology is non-invasive without rundown, even over several days, repetitive dynamic measures are possible over physiologically meaningful time spans. This allows to test a series of physiological relevant parameters in a dynamical fashion, as opposed to simple glucose-induced increase of islet activity [15, 18, 22]. To rank the performance of donor islets according to SP frequencies, we established a number of criteria and ensuing testing scenario. The score rankings were established prior to actual recordings ranging from CHIP-scores 1 to 6, reflecting the least physiological performance, i.e., glucose insensitivity, to the most physiological performance, i.e., glucose concentration dependency and biphasic activity pattern [15, 17] (see Figure 1C; Supplementary Table S1). The criteria applied here are based on well-known islet physiology [14, 26]: at low glucose, islet β-cells show no or only minor spontaneous activity; the neurohormone epinephrine inhibits β-cell activity, an important feature during physical activity or stress; increasing levels of glucose over its physiological range considerably enhances electrical activity and in the best case, this electrical activity is biphasic; the stimulation by glucose is further augmented postprandially by the incretin hormones, such as GLP-1, at physiological levels of 50 pmol/L and less [14, 15, 22]; and finally, sulfonyl-urea drugs such as glibenclamide stimulate islet β-cells independent from glucose via pharmacological closure of K_ATP_ channels. The absence of glucose, GLP-1 or glibenclamide induced activity was ranked as least performant with a score of 1 (Figure 1C, red numbers) and those islets were not further investigated. If at least glibenclamide or GLP-1 effects were observed, a score of 2; if responses to GLP-1 and to the stress hormone adrenalin were observed, a score of 3; if glucose concentration dependency was evident only at high glucose concentrations (15 mM vs. 1 mM), a score of 4 was attributed; if glucose concentration dependency was observed over the physiological range of 5.5 mM–11 mM glucose, a score of 5 was given; finally, if biphasic glucose-induced activation was observed, the (highest) score of 6 was assigned. Islets were consequently tested for basal and non-β cell activity using adrenaline known to inhibit β- and stimulate α-cells (Figure 1C; ➊,➋), responsiveness to elevated glucose (Figure 1C; ➌), glucose concentration dependency (Figure 1C; ➍), biphasic activity at physiological glucose concentrations (Figure 1C; ➎), and the effects of drugs such as the sulfonylurea glibenclamide or hormones (GLP-1, adrenaline) on glucose-induced activity (Figure 1C; ➏,➐,➑).

Two MEA recordings are given in Figure 1D (black traces) as examples of lowest (CHIP-score 1; maximum frequency 0.095 ± 0.014 Hz) and highest CHIP-score (CHIP-score 6; maximum frequency 0.409 ± 0.034 Hz) and their corresponding β−scores are provided in Figure 1D. Recordings from electrodes not covered with islets were provided (Figure 1D, blue traces) and show the high signal-to-noise ratio of the MEA approach. Recordings with the lowest CHIP-score (1; upper panel) showed neither clear glucose-dependency nor any effect of GLP-1 or even glibenclamide and were not submitted to further tests. Recordings with the highest CHIP-score of 6 (lower panel) passed successfully steps 1 (as shown), as well as 3 and 4 (traces not shown) and exhibited a clear biphasic increase in SP frequency in step 5 (as shown). The peak of the first phase occurred after 5 minutes, in-line with reported electrical behavior of human islets [14, 15, 17] and exhibited even typical 5–10 min oscillations in the second phase.

**FIGURE 2:**
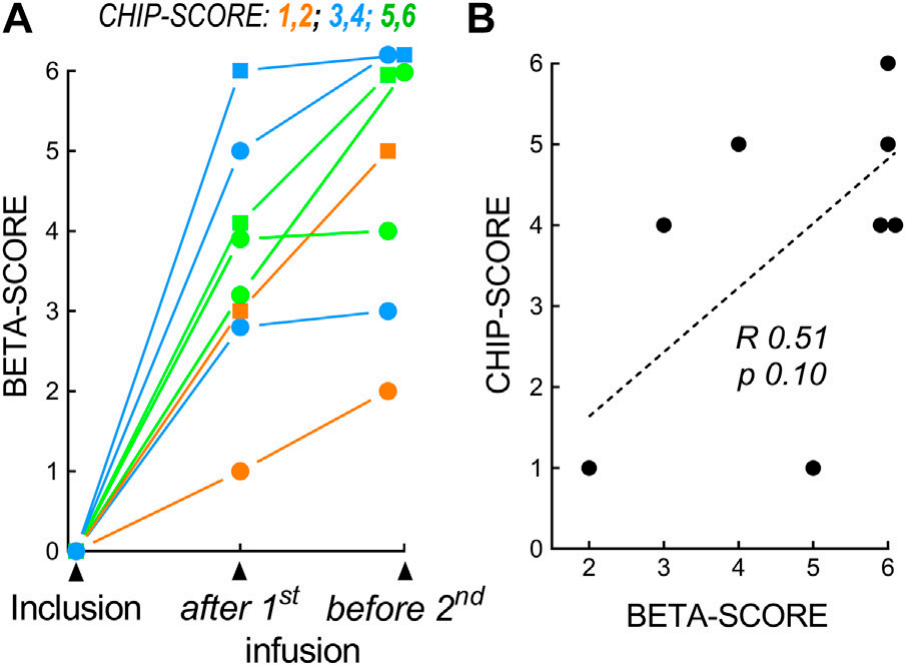
Correlation of CHIP-scores and β-scores. **(A)** β-scores at inclusion, after the 1st islet infusion, and before the 2nd islet infusion. Three subgroups were identified in relation to the β-score and CHIP-score. Orange, CHIP-score 1 to 2, no detectable β-cell activity; blue, CHIP-score 3 to 4, moderate glucose dependency; green, CHIP-score 5 to 6, excellent activity. **(B)** Correlation (R, Spearman) for CHIP-Score and β-score (before 2nd infusion).

The Islet CHIP study included 8 recipients from the STABILOT clinical islet transplantation study (4 men and 4 women; mean diabetes duration, 34 ± 11 years; Table 1; Supplementary Table S1). The evolution of primary graft function evaluated using the β-score prior to the second infusion is shown in Figure 2A. Groups of CHIP-score vs. β-score are apparent: patients who improved rapidly after the 1st infusion and further before the 2nd infusion (Figure 2A, green), patients who improved only partially after the 1st infusion (Figure 2A, blue), and patients who changed little after the 1st infusion and progressed little afterwards (Figure 2A, orange). There was a correlation between the β-score established prior to the second islet infusion and the CHIP-score (Figure 2B; ρ = 0.51, *p* = 0.1), as well as between HbA_1c_ levels and CHIP-score; ρ = −0.556, *p* = 0.08) but statistical significance was not attained in either case. Islet purity, GSIS or total amounts of insulin secreted did not correlate with the β-score, HbA_1c_ level, or CHIP-score as reported previously. CHIP-score did not correlate with main donor criteria such as age, sex, BMI, cause of death, warm or cold ischemia time. These latter parameters as well as volume or amount of IEQ infused were also not correlated with β-scores.

## Discussion

The most relevant score in terms of success is given by the β-score and final clinical outcome will evidently depend on numerous parameters including recipients’ characteristics. It is thus reasonable to expect that donor organ quality may only be one of the many factors influencing the clinical outcome [27]. Obviously, determining the quality of the main therapeutic agent remains a major issue as in any clinical intervention. Function of human islet transplanted in nude mice correlates well with clinical outcomes. Although this highlights the importance of quality of islets to be transplanted, the read-out of this assay is only retrospective [11]. New approaches to quality control are required and they may also provide means for better comparison of results between transplantation cohorts in view of the diverging criteria applied in donor selections [28].

Previously, a number of islet parameters were tested for their predictive value in animal transplantation studies [29]. Mitochondrial markers have been reported as significant indicators in the case of allo- or auto-transplants [30, 31]. However, islets were generally of lower purity, and the clinical endpoints used were either insulin dependency or independence, which is quite different from the currently used scaled β-score. Studies on the size of islets used for transplantation have not shown any correlation with clinical outcome [32]. Investigation of donor long noncoding RNA repertoire revealed that *MALAT1* expression predicts the quality of human islets prior to their isolation [33] but a potential correlation of this signature with clinical outcomes has not been published.

Similar to our study, insulin secretion of donor islets prior to transplantation has been reported to correlate poorly, if at all, with outcome in animal transplantation studies [29, 34]. In contrast to GSIS, the CHIP-score described here relies on combining a proven technology [15, 17, 18, 22, 23] with parameters such as a range of glucose concentrations, as compared to a single concentration of high glucose, relevant hormones and direct assessment of K_ATP_-channel function, central to islet activity. Moreover, the potential clinical relevance of the electrical signals recorded here by MEAs is underscored by the observation that they can be used in an FDA approved simulator of human metabolism of patients afflicted by type 1 diabetes to control *in silico* glucose homeostasis via insulin delivery [18, 19]. For those reasons the static evaluation of insulin secretion by GSIS may not provide sufficient details on islet function, including physiologically relevant parameters, such as biphasic activity and β-cell coupling [14]. Additional parameters as used here in the electrophysiological characterization (hormones and detailed glucose concentration dependency), may *per se* also be determined in classical dynamic secretion assays. However, this would considerably increase the workload and costs as compared to an automated electrophysiological analysis. Moreover, dynamic measurements of insulin release do not inform *per se* about the important physiological parameter of islet β-cells, which is coupling. Finally, the MEA technology required is fully compatible with the expertise of a clinical laboratory.

As expected from a small sample sized pilot study, the limitation of our study is the absence of statistical significance, despite a good correlation, that may also reflect the influence of multiple confounding clinical factors.

In conclusion, to the best of our knowledge, this pilot study is the first to correlate donor islet functional quality and clinical outcomes prior to human allotransplantation. Biomimetic potency tests have been strongly advocated for islet transplantation, and recent progress in islets-on-chip may provide solutions [12, 35]. Our observations indicate a potential usefulness of our islets-on-chip system presented here in evaluating islets before grafting and might consequently improve clinical outcomes. The approach used here may also be developed as microfluidic device thus further reducing the number of islets required [15, 23, 36, 37]. In the long run, such a system might also qualify for evaluation of stem cell-derived pseudo-islet organs prior to their implantation [12].

## Data Availability

The raw data supporting the conclusion of this article will be made available by the authors, without undue reservation.

## Abbreviations

cAMP, cyclic AMP; GLP-1, Glucagon-like peptide 1; GSIS, Glucose-induced insulin secretion; HbA_1c_, Glycated hemoglobin; MEA, Multielectrode array; SP, Slow potentials.
