# Supplementary material for: Islets-on-Chip: A Tool for Real-Time Assessment of Islet Function Prior to Transplantation
Source: Transpl Int. 2023 Oct 11;36:11512. doi: 10.3389/ti.2023.11512 (PMC10598278; doi:10.3389/ti.2023.11512)
Supplement: Supplementary file 1 [file Table1.DOCX]

| **Checklist for Reporting Human Islet Preparations Used in Research**  Adapted from Hart NJ, Powers AC (2018) Progress, challenges, and suggestions for using human islets to understand islet biology and human diabetes. Diabetologia <https://doi.org/10.1007/s00125-018-4772-2>. | |
| --- | --- |
| **Manuscript DOI:** | |
| **Title: Islets-on-chip: A tool for the real-time assessment of islet function prior to transplantation** | |
| **Author list:** Matthieu Raoux, Sandrine Lablanche, Manon Jaffredo, Antoine Pirog, Pierre-Yves Benhamou, Fanny Lebreton, Anne Wojtusciszyn, Domenico Bosco, Thierry Berney, Sylvie Renaud, Jochen Lang, Bogdan Catargi | |
| **Corresponding authors:** Jochen LANG, Bogdan CATARGI | **Email address:**  [**Jochen.lang@u-bordeaux.fr**](mailto:Jochen.lang@u-bordeaux.fr) [**bogdan.catargi@chu-bordeaux.fr**](mailto:bogdan.catargi@chu-bordeaux.fr) |
