# Supplementary material for: Islets-on-Chip: A Tool for Real-Time Assessment of Islet Function Prior to Transplantation
Source: Transpl Int. 2023 Oct 11;36:11512. doi: 10.3389/ti.2023.11512 (PMC10598278; doi:10.3389/ti.2023.11512)

**Supplemental Table 2: Characteristics of the different CHIP SCORES.** Note that islets that did not respond to typical islet stimuli (such as 15 mM glucose or 15mM glucose with glibenclamide) by increases in SP frequency, were not further analyzed. YES, effect is present; NO, no effect observed. The test step numbers (1 to 8) are the same as in Figure 1C.


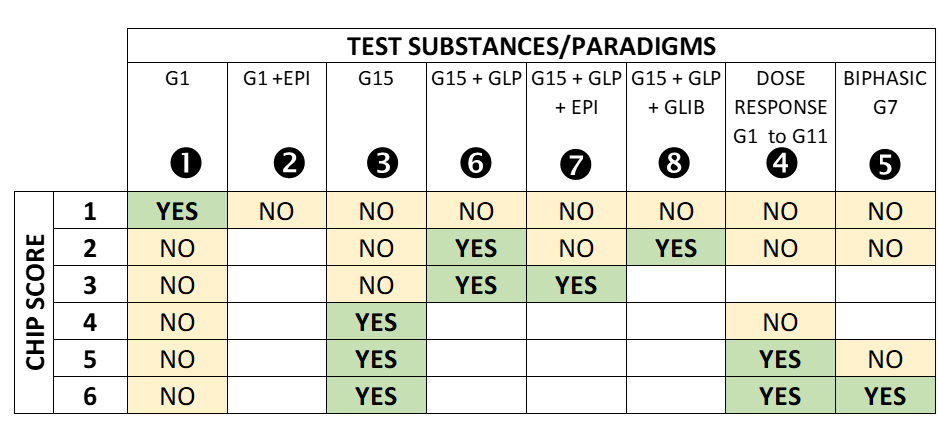

Supplement: Supplementary file 2 [file Table2.DOCX]
